# Practice points for lymphoedema care in low- and middle- income countries developed by nominal group technique

**DOI:** 10.1186/s12913-023-09786-w

**Published:** 2023-07-08

**Authors:** Eric Torgbenu, Tim Luckett, Mark Buhagiar, Jane L. Phillips

**Affiliations:** 1 IMPACCT (Improving Palliative, Aged and Chronic Care Through Clinical Research and Translation), Faculty of Health, University of Technology Sydney, Sydney, NSW Australia; 2 Catholic Diocese of Parramatta, Parramatta, NSW Australia; 3 School of Nursing, Queensland University of Technology, Brisbane, Australia

**Keywords:** Lymphoedema, Practice points, Guidelines, Nominal group technique, Low- and middle-income countries

## Abstract

**Background:**

Lymphoedema is a common, distressing, and debilitating condition affecting more than 200 million people globally. There is a small body of evidence to guide lymphoedema care which underpins several lymphoedema clinical practice guidelines developed for high-income countries (HIC). Some of these recommendations are unlikely to be feasible in low-resource settings.

**Aim:**

To develop practice points for healthcare workers that optimise lymphoedema care in low- and middle-income countries (LMIC).

**Methods:**

A nominal group technique (NGT) was undertaken to gain consensus on which content from HIC guidelines was important and feasible to include in practice points for LMIC, and other important advice or recommendations. Participants included experts, clinicians, and volunteers involved in lymphoedema care in LMIC. The NGT followed five key stages: silent ‘ideas’ generation, round-robin rationale, clarification, refinement and verification. The first, fourth and fifth stages were completed via email, and the second and third during a video meeting in order to generate a series of consensus based prevention, assessment, diagnosis, and management of lymphoedema in LMIC practice points.

**Results:**

Of sixteen participants invited, ten members completed stage 1 of the NGT (ideas generation), of whom six contributed to stages 2 (round-robin) and 3 (clarification). All those who completed stage 1 also completed stages 4 (refinement) and 5 (verification). Practice points unanimously agreed on included Complex Decongestive Therapy (CDT) and good skin care, with management to be determined by lymphoedema stage. For podoconiosis-endemic areas, the use of socks and shoes was identified as very important in the prevention of non-filarial lymphoedema and other lymphoedema-causing conditions. Participants indicated that diagnosing lymphoedema using the lymphoscintigraphy and Indocyanine green (ICG) fluorescent lymphography was not possible due to unavailability and cost in LMIC. Surgical procedures for lymphoedema management were unanimously eliminated due to the unavailability of technology, limited workforce, and expensive cost in LMIC.

**Conclusion:**

The consensus-based practice points generated by this project provide healthcare workers with guidance on caring for people with lymphoedema in LMIC. Further development of workforce capacity is needed.

**Supplementary Information:**

The online version contains supplementary material available at 10.1186/s12913-023-09786-w.

## Background

Lymphoedema is a chronic and debilitating condition, often referred to as the “forgotten complication” because it can be mistaken for oedema, venous insufficiency or heart disease [1–3]. Globally, more than 200 million people are affected by lymphoedema [4], the majority of whom live in low- and middle-income countries (LMIC). The condition impacts negatively on quality of life, interferes with activities of daily living and ability to work, and is traumatic for the individuals affected and their families, lymphoedema alters their “sense of identity” and reduces their ability to participate in social events [5, 6].

Compared to people in high-income countries (HIC), those living in LMIC are often diagnosed at a later stage and with more advanced forms of lymphoedema [7]. They also have fewer treatment options, which increases their risk of developing complications and poorer health [8]. Unlike in HIC where cancer treatment is the major cause of lymphoedema, the most common cause of lymphoedema in LMIC is infectious lymphatic filariasis, while others suffer from the “swollen limb syndrome” [9, 10]. The World Health Organisation (WHO) reported that there has been a rapid reduction (74%) in lymphatic filariasis since the beginning of the ‘Global Programme to Eliminate Lymphatic Filariasis’ in 2000, limiting the impact of the disease in most areas [9, 11]. However, lymphoedema from other causes such as cancer, podoconiosis, idiopathy and trauma remains a major problem.

A recent systematic review [12] identified ten lymphoedema guidelines for HIC but only one for LMIC: the WHO *Wound and Lymphoedema Management* [13], which focuses exclusively on lymphoedema caused by infectious filariasis and wounds, and has very limited content on diagnosis and assessment. Unfortunately, recommendations in HIC guidelines are not always transferable to LMIC, especially when access to specialist services is limited and healthcare workers lack high levels of expertise to provide optimal care [14]. In many LMIC, awareness of lymphoedema by the general public is limited and those affected are often stigmatised due to cultural beliefs [15]. Finally, financial constraints for many people in LMIC negatively affect their capacity to pay for medical supplies and professional support.

In the absence of robust evidence to guide the prevention, identification and management of lymphoedema in LMIC, ‘practice points’ can fill this gap and are often highly valued by health workers. Practice points are sets of recommendations that are developed through expert opinion when evidence is insufficient or outside the scope of systematic reviews [16].

The current research aimed to develop practice points for healthcare workers that optimise lymphoedema care in LMIC.

## Methods

### Design

A nominal group technique (NGT) using a formal consensus process, was adopted as an interpretative method for generating a series of lymphoedema care practice points [17, 18].

A NGT is a technique of interviewing where group members independently provide ideas without interrupting each other [19]. The technique emphasizes considering all participants' views equally and enhancing group dynamics [18]. The NGT has been used in the past to evaluate priorities for health services, and formulate policies both in treatment of health conditions including cancer and in other health settings [20, 21]. The group deliberation and discussion of specific topics used in NGT help to minimise misunderstanding on a topic and provide highly suitable responses [20]. Where evidence is limited like in the area of lymphoedema care in LMIC, NGT consensus provides a useful method in developing clinical practice points.

### Participants and setting

To be eligible to contribute, participant needed to be: a) expert on the diagnosis, assessment and/or management of lymphoedema from any cause, as denoted by authorship on a relevant journal article and/or b) health professional or caregiver in a World Bank Group [22] LMIC in the ‘paid’ or voluntary capacity.

### Recruitment strategy

Participants were sampled using two non-probabilistic methods: purposive and snowballing methods [23, 24]. Initial approach was via email invitation to authors of relevant journal articles identified by a systematic review of lymphoedema studies in LMIC [25], personal and professional networks, and through organizations with an interest in lymphoedema care in LMIC, as well as to participants of previous research on lymphoedema care in LMIC [26].

Email invitations to individuals were followed up with a reminder after a week, to ensure that people had ample opportunity to respond without unduly ‘pestering’ them. A non-response to the follow up email was considered a decline to participate [27].

Participants who completed the first stage on the idea generation survey via email were invited to participate in stages 2 to 5.

### Data collection

The NGT consensus was used to identify which key topic areas should be prioritised for simple and practical guidance on lymphoedema care for clinicians in LMIC. This work followed an adapted version of McMillan et al*.* [28] five NGT stages, as summarised below:

### Stage 1

Stage 1 (silent generation) is an idea generation stage. Participants were emailed a list of topic areas from the HIC guidelines asking them to individually reflect on which ones they think are most important and feasible for LMIC as well as to add any further ideas they have for such guidance. Each participant returned their ideas to the first investigator (ET) for him to identify areas of agreement and disagreement.

### Stage 2

In a ‘round robin’ stage, participants attending a meeting were asked to explain the rationale for their ‘top choice’ for inclusion in the clinician guidance. If a participant’s top choice had already been put forward by someone else, (s)he was invited to choose another. The round robin continued until everyone’s choices were exhausted. No group discussion or commentary by the facilitator occurred at this stage. This stage and stage 3 were completed at a video meeting facilitated by the second author (TL), with notes taken by ET.

### Stage 3

The next stage focused on clarification of ideas via group discussion, in which participants were asked to identify areas of overlap or synergy between the selected topics to create themes, ensuring that the list was coherent and not repetitive. At this stage, participants could also agree to exclude or alter ideas, as well as generate new ideas. Feasibility in terms of cost for each recommendation was appraised subjectively by NGT participants based on their own experience rather than using a pre-specified monetary threshold.

### Stage 4

In the fourth stage (refinement), the group choices from the list created at Stage 3 for each domain of the guidance document were sent via email to participants for verification. Participants were allowed to comment on the guidance document individually rather than as a group to ensure all participants’ opinions were captured. Where a practice point was suggested by a participant that was additional to those in the HIC guidelines, a Medline database search was conducted to check the evidence available either in support or against the suggestion. Where evidence was considered favourable or absent, the suggestion was put forward for discussion and consensus. Consensus was defined as a 100% agreement from all participants.

Recommendations were graded based on the systems as reported by the *International Lymphoedema Framework* [29]. Levels of evidence for each recommendation were defined by the relevant guideline(s) from which it was sourced. All of the guidelines except two [30, 31] used the grading system described by the International Lymphoedema Framework [27]:

- Grade A—clear research evidence obtained from meta-analysis or systematic review of randomized controlled trials;
- Grade B—limited supporting research evidence; and
- Grade C—experienced common sense judgement (consensus) [29].

### Stage 5

In the final stage (verification), results from the fourth Stage were presented back to the group for discussion and consensus, again via email. Where a participant suggested a review, the new suggestion was made and returned via email to all participants for another round of verification until there were no more additional information. ET synthesised all the feedbacks and identified areas of agreement and consensus and presented the practice points for lymphoedema prevention, assessment, diagnosis and management.

### Analysis

Analysis of Stage 1 data involved simple counts of the number of participants identifying each topic as important and/or feasible. Analysis for Stages 3 and 5 used a simple qualitative approach, with themes inductively derived from participant discussion and verified with participants then modified according to feedback [32]. At the end of Stage 4, descriptive statistics of median and range were used to indicate consensus among participants for each topic based on survey results [33, 34].

### Ethical considerations

Projects building consensus among experts are not required to obtain formal ethical approval according to policy at the University of Technology Sydney provided participants agree to be identified on reports. It was made clear during recruitment that participation was voluntary for ethical consideration, and that completion of Stage 1 and/or attendance at the meeting for Stages 2 to 5 would be taken as evidence for consent. Participation included an understanding that names would be published, in accordance with common practice for consensus studies [35]. However, each participant’s responses to the survey remained anonymous. They also reserved the right to discontinue participation at any time. These terms were repeated at commencement of the meeting.

The NGT consensus was conducted between December 2021 and May 2022.

Out of the sixteen participants invited, ten completed Stage 1 (idea generation), and six attended the meeting to complete Stages 2 (round-robin) and 3 (clarification) and reach a consensus on contents for inclusion in the practice points. Participants were representatives from the disciplines of physiotherapy (*n* = 4), nursing (*n* = 1), and medicine (*n* = 1). All participants had more than ten years of experience of providing lymphoedema care in LMIC. All experts also had advanced education (masters or doctorate), and were affiliated to either a higher academic, research and/or health institution. The experts had expertise in providing care in the following LMIC; Bangladesh, Brazil, Cooks Islands, Egypt, Ethiopia, Ghana, Haiti, India, Ivory Coast, Papua New Guinea, and the Solomon Islands.

### Consensus

Consensus was reached on twenty-five of the thirty-three items included in stages 2–5 of the NGT. The highest level of consensus was reached for lymphoedema prevention, diagnosis and non-surgical management (Refer to Tables 1 and 2 for outcome of Stage 1 of the NGT consensus), while no consensus was achieved for the surgical management of lymphoedema in LMIC. There were very low levels of support for promoting costly preventative, diagnostic or surgical interventions that required specialist equipment or capabilities. Diagnostic techniques such as indocyanine green, bioimpedance spectroscopy and lymphoscintigraphy along with the surgical procedures recommended in HIC, such as liposuction, lymphovenous anastomosis and vascularised lymph node transfer were all excluded from the lymphoedema practice points for LMIC.

**Table 1:** Outcome of Stage 1 of the NGT consensus on lymphoedema prevention and diagnosis

Key Topic Area	Important/feasible for LMIC guidance	Overall synthesis	
	Yes (*N* = 10)	*If not, why not*	*Main considerations*
**A) Lymphoedema Prevention**			
1. Lymphoedema education	10	None	*Significantly important, using simple language that is presented with more visual illustrations, including self-hygiene as part of the care*
2. Promoting self-management	10	None	*Significantly important, and consider partner and family if appropriate*
3. Morbidity control through prevention and treatment	10	None	*Significantly important, and should include “early detection”*
**B) Lymphoedema Diagnosis**			
4. Circumferential measurement using tape measure	10	None	*Requires minimal technique; readily available and quick to use. Patients/families can be taught to measure their arms in regular intervals for monitoring. However, a standard and consistent method need to be used*
5. Volumetric measurement	8	*Not widely available, and it poses the risk of cross-infection if infection control measures are not strictly followed; consider availability of portable water; too messy with water usage, application for calculating volume measure recommended*	*Probably the most available method of measurement in some places*
6. Bioimpedance spectroscopy	6	*Not readily available; probably least available tool to diagnose in LMIC due to cost and lack of training*	*Increasingly available in some low- and middle-income countries*
7. Indocyanine green lymphangiography	5	*Unavailable and costly; not necessary except in elective surgery; least available due to cost and lack of training*	*If available, will be very helpful to appreciate individual flow patterns; necessary to consider availability of human resource and cost*
8. Lymphoscintigraphy	3	*Unavailable and cost; unavailability of human resource; most patients cannot afford it; lack of expertise*	*Might not be available*
9. Magnetic resonance lymphangiography	3	*Unavailable and costly; least available tool to diagnose lymphoedema in LMIC due to cost and lack of training*	-

*LMIC* Low- and middle-income countries; *HIC* High-income countries

“If not, why not?”? – subjective appraisal of importance or feasibility of topic areas by participants

Numbers listed in column 2 refer to the level of consensus on a topic

**Table 2:** Outcome of Stage 1 of the NGT consensus on surgical and non-surgical management

Key Topic Area	Important/feasible for LMIC guidance	Overall synthesis	
	Yes (*N* = 10)	*If not, why not*	*Main considerations*
**A) Non-surgical management of lymphoedema**			
1. Skin care	10	-	*Simple and basic. However, materials and tools need to be available*
2. Compressional therapy (including garmenting)	10	*Consider high cost of bandages and non-availability of garments*	*Need deep training on distorted real size models, to learn bandaging huge oedematous areas in the intensive phase of treatment. Need in-depth clinical guidelines to choose between different compression tools in the conservative phase of treatment; can use local materials*
3. Exercise prescription	10	-	*Very necessary, considering various stages of condition. It should include pelvic floor and core exercises as routine for people with genital lymphoedema*
4. Psychosocial rehabilitation considering the family support systems	10	*Sometimes, it adds financial burden on the patients and where necessary, may be eliminated*	*Very critical and important in low- and middle-income countries; it should be provided by informal caregivers. Also, required in destigmatising the disease*
5. Quality of life improvement strategies	10	*If it adds financial burden on the patient, it could be cancelled*	*It requires quality assessment tools*
6. Cellulitis management	10	-	*Important to identify and intervene early. Patients were often told to contact the treating oncology settings for management by the first contact healthcare practitioner. Therefore, information related to this condition must be explained to the lymphoedema patients; very important due to high bacterial load of the skin & bad hygiene, and could benefit from new evidence (compression therapy management)*
7. Weight management techniques/ advice	10	-	*It should include the importance of healthy diet and should be included in education. Advice on obesity needs to be based on the criteria specific to the intended population on which the intervention is initiated. Normal BMI: 18.0–22.9 kg/m2, Overweight: 23.0–24.9 kg/m2, Obesity:* > *25 kg/m2*
8. Referral to appropriate specialists where indicated	10	*Absence of specialists; consider availability as most areas have limited specialists*	*Often the referral is unnecessarily delayed due to a range of reasons. A guideline with a specific referral pattern may be helpful*
18. Complete Decongestive Therapy—Manual lymphatic drainage	9	*Lack of evidence*	*Therapists require training*
9. Antimicrobial therapy	8	*It is not routinely used in practice. For low- and middle-income countries, it is important to consider cost. Very necessary to consider availability. Some places must be have. For instance, penicillin is readily available for patients in Uganda*	*Quite important in controlling bacterial infections in people with infected wounds and might not be in lymphoedema *per se
10. Teaching of firm self-massage techniques	8	*Cost in training and danger if not done appropriately*	-
11. Diuretics	6	*Should only be used if required*	*Only if appropriate to use for example, in reducing volume overload in cases with cardiac heart failure*
12. Medical therapy—Benzopyrones	3	*Not enough good quality evidence for effectiveness; Not widely used; not routinely used in practice. It is hepatoxic and lacks quality evidence and therefore should not be recommended*	-
**B) Surgical management of Lymphoedema**			
13. Liposuction	1	*Not available in LMIC; and should be reserved for complex cases. It has poor quality of evidence and may be costly*	*Consider availability of human resources (plastic surgeons) and cost; Surgical procedures should only be reserved for complex or advanced cases. Do not suggest debulking. Procedures should only be done by specially trained providers and risks are high. Not readily available even in many HIC and it appears to only work in carefully selected patients*
14. Vascularised lymph node transfer	0	*Not available in LMIC, absence and inexperienced specialists; usually patients are at stages 2–3; microsurgeries are not applicable; poor quality evidence of effectiveness; high cost; and only works in carefully selected patients*	*Not readily available even in many HIC and it appears to only work in carefully selected patients; Surgeons may not be skilled enough and procedure may be too expensive*
15. Lymphaticovenous anastomosis	0	*Not available in LMIC; not indicated to all patients; limited and inexperienced specialists; poor quality evidence of effectiveness; and not indicated to patients presenting in stages 2–3; and microsurgeries are not applicable*	*Not readily available even in many HIC and it appears to work in carefully selected patients; Surgeons may not be skilled enough and procedure may be too expensive*

*LMIC* Low- and middle-income countries, *HIC* High-income countries

“If not, why not?”? – subjective appraisal of importance or feasibility of topic areas by participants

Numbers listed in column 2 refer to the level of consensus on a topic

## Results

Across prevention, diagnosis, assessment and management, eighteen practice points were recommended, each with accompanying key areas for the simple, practical guidance, as summarised below and detailed in Table 3. A list of societies and organisations guidelines used in the development of the practice points have been provided in Supplementary Table 1 with their web pages.

**Table 3:** Practice points: lymphoedema prevention, diagnosis, non-surgical and surgical management in LMIC

Number	Recommendation or Practice Point
**A) Prevention**	
i) **Awareness creation**	
• Effective identification of people at risk of developing lymphoedema through creating awareness about causes of lymphoedema and associated risks factors, implementation of preventive strategies, as well as self-monitoring. (GRADE C), ILF [29] ONS [30] ISL [8] • Patient and carers should have early active involvement in the management of lymphoedema including appropriate information on skin care. (GRADE B), ILF [29]	
***Practice Point 1:*** Raise awareness regarding lymphoedema and the risks posed by surgery and severe infections; and promoting diligent skin care and optimal treatment of all comorbid conditions (e.g., hypertension, diabetes, obesity, and/or heart conditions). (*Consensus through NGT Meeting*)	
ii) **Multidisciplinary team approach**	
• Lymphoedema care requires a multidisciplinary team approach requiring healthcare workers including: general practitioners (GPs), surgeons, physicians, physiotherapists, occupational therapists, nurses, dietitians, podiatrists, counsellors and service managers, among others. (*Consensus*) ACI [36] ILF [29]	
***Practice Point 2:*** Ensure healthcare workers are aware of any available specialist services and the need for a multidisciplinary team (MDT) approach to lymphoedema care. (*Consensus through NGT Meeting*)	
iii) **Promoting skin care**	
• Individual with lymphoedema should be provided with skin care education as well as proper maintenance of appropriate skin care practices. (*Consensus*) ACI [36] • Minimising the risk of cellulitis/erysipelas by treating any condition associated with lymphoedema. (GRADE B), ILF [29]	
***Practice Point 3:*** Promote diligent skin care and treating other comorbid conditions like hypertension, diabetes, obesity, or heart conditions. (*Consensus through NGT Meeting*)	
iv) **Protective clothes**	
• To reduce the risk of deterioration or lymphoedema, people with lymphoedema should wear comfortable, supportive shoes, and wear prophylactic compression sleeves, where indicated. (*Consensus*) ILF [29]	
***Practice Point 4:*** Promote the use of socks and shoes where there is a risk of podoconiosis. (*Consensus through NGT)*	
• An accurate assessment including filarial antigen test is recommended to detect infection by *Wuchereria bancrofti* mainly for persons living or have visited lymphatic filariasis endemic area. (GRADE C) ILF [29]	
***Practice Point 5:*** Initiate laboratory tests for lymphatic filariasis. (*Consensus through NGT)*	
vi) **Mosquito control**	
• Controlling the vector, mosquito will prevent the risk of lymphatic filariasis infection transmission. Mosquito nets should be used in lymphatic filalriasis endemic areas, and caregivers and people with lymphoedema should be informed on mosquito control strategies. (GRADE C) ILF [29]	
***Practice Point 6:*** Promote mosquito control to prevent filarial lymphoedema. (*Consensus through NGT)*	
**B) Assessment**	
i) **Signs and symptoms**	
• Check for changes in the skin. (*Consensus,* ILF [8]) (Grade A), IUP [37] • Signs and symptoms of heaviness, swelling, tight clothing, and pitting should be considered. (*Consensus*) APTA [38] • Identifying any other health conditions that might affect or be affected by lymphoedema. (*Consensus*) ILF [29]	
***Practice Point 7:*** Assess changes in the skin, including dryness, pigmentation, agility, warmth, cellulitis, scars, chronic wound and ulcers, deepened skin folds, skin breakdown and Stemmer sign; refer for comprehensive assessment by trained staff where available. (*Consensus through NGT)*	
ii) **Objective assessment**	
• Establish a baseline measurement through volume measurement, and take circumferential measurement and compare affected limb with unaffected (Grade B) IUA [39]; *(*Consensus, ACI [36] and ISL [8]) • Perform Stemmer’s test. Stemmer’s sign is positive when a thickened skin fold at the dorsum or toe cannot be lifted up or is difficult to lift. (Consensus, CREST [40] and ILF [29])	
***Practice Point 8:*** Take circumferential measurements of affected limb and compare with the unaffected using a tape measure (check for fitting clothes, and changes in limb size) and/or volume measurement. (*Consensus through NGT*)	
iii) **3D Scanning**	
• Using 3D scanning technology • Using mobile applications such as the LymhaTech and scan photos via WhatsApp [41] and other appropriate applications. There is no effective method for measuring oedema of the head and neck, breast, trunk or genitalia. It is recommended to take a digital photography to record and monitor facial and genital lymphoedema. (Consensus) ILF [29]	
***Practice Point 9:*** Assessment of lymphoedema using 3D Scanning devices, as well as scanning photos through WhatsApp and other appropriate applications. (*Consensus through NGT*)	
iv) **Telemedicine**	
• Telemedicine and telehealth should be used in areas of limited resources to improve local provider’s capacity. (Consensus). ACI [36]	
***Practice Point 10:*** Practice telemedicine using computer applications using emails, and WhatsApp. (*Consensus through NGT*)	
v) **Staging**	
• Indicate the stage of condition as per the ISL classification	
Note: ◦ Stage 0—A subclinical state where swelling is not evident despite impaired lymph transport. This stage may exist for months or years before oedema becomes evident ◦ Stage 1—This represents early onset of the condition where there is accumulation of tissue fluid that subsides with limb elevation. The oedema may be pitting at this stage ◦ Stage 2—Persistent pitting oedema is manifest and limb elevation alone does not reduce swelling ◦ Late Stage 2—Persistent swelling, there may or may not be pitting as tissue fibrosis is more evident ◦ Stage 3—The tissue is hard (fibrotic) and pitting oedema may be absent. Skin changes such as thickening, hyperpigmentation, increased skin folds, fat deposits and warty over growths develop. The most severe changes are also known as elephantiasis. (Consensus) ISL [8]	
***Practice Point 11:*** Indicating the stage of the disease condition in accordance with the ISL staging. (*Consensus through NGT*)	
vi) **Questionnaires**	
• Assess the impact of lymphoedema on daily lives of people with lymphoedema using questionnaires. (GRADE C) ILF [29]	
***Practice Point 12:*** Use questionnaires to understand the impact of lymphoedema on person’s daily life. (*Consensus through NGT*)	
**C) Diagnosis**	
i) **History taking and physical examination**	
• Begin diagnosis with a Doppler scan. (Consensus)ACI [36]	
• Take history and perform physical examination; ruling out secondary conditions or vascular problems using Doppler and magnetic resonance imaging (MRI). (GRADE A) IUA [39]	
• Use Bioimpedance spectroscopy; and make diagnosis of any arterial problems. (Consensus) ISL [8]; (Level II) QH [42]	
• Perform immunochromatographic card test (ICT) or indocyanine green (ICG) to rule out lymphatic filariasis in people with podoconiosis. (*Consensus through NGT*)	
***Practice Point 13:*** Compare records of tape measurement, physical examinations, Doppler scan, photos in both unilateral and bilateral lymphoedema, where necessary compare affected to unaffected limbs coupled with subjective assessment to make diagnosis. (*Consensus through NGT*)	
***Practice Point 14:*** For podoconiosis, request ICT to rule out filariasis or Indocyanine green (ICG) if available. (*Consensus through NGT*)	
**D) Management**	
i) **Key management techniques**	
• Lymphoedema management should be based on the severity and extent of the condition	
At subclinical stage (stage 0), management should involve: a) weight management, diet and skin care; b) individualized aerobic exercise programs, and specific treatment regimen; (GRADE A) APTA [43] and c) prescription of other treatment including education, self-massage, and compression garments (GRADE C)*,* APTA [43]; IUP [37]; JLSG [44]	
• Management at early onset (stage 1) includes:	
a) Where there are early signs and/or symptoms of lymphoedema, recommend compression garment and instruct on exercise regimen as well as provide education as first-line treatment. (GRADE A), APTA [43]	
b) Elevation, specialised exercise, appropriate compression sleeve to wear for at least 12 h per day where indicated *Knowledge gap,* ONS [30]; *Level 2b,* BPG [31]	
c) Provision of ongoing education for care, managing pre-morbid conditions, and weight management strategies (GRADE C) JLSG [44]	
• Where there is persistent oedema (stage 2 and above), management should include:	
a) Teach self-bandaging to patient, and prescribe complex decongestive therapy (including compressive therapy and bandaging which require support and follow-up); (Grade B), IUP [37]; (Graded conditional), ONS [30]; (Grade A), APTA [43]; and (Level 2a), BPG [31]	
b) prescribe cuff/muscle pumps where available and manual lymphatic drainage; (Grade B), ILF [29]; (Grade C), IUP [37] and	
c) for an optimal long-term reduction in volume, monitoring volume changes and providing frequent follow-up care (GRADE C), APTA [43]	
***Practice Point 15:*** Meticulous skin care, exercise, modified lymphatic massage, manual lymphatic drainage, and compression therapy (wraps) based on the stage of the condition. (*Consensus through NGT*)	
***Practice Point 16:*** Perform a functional, aggressive physiotherapy to improve hand/finger movements for people with severe fibrosis. (*Consensus through NGT*)	
***Practice Point 17:*** Ensure foot hygiene and skin care at all stages of lymphoedema. (*Consensus through NGT*)	
***Practice Point 18:*** Include pelvic floor exercises for people living with genital lymphoedema. (*Consensus through NGT*)	

*ACI* Agency for Clinical Innovation, *APTA* American Physical Therapy Association, *BPG* Best Practice Guideline, *CREST* Clinical Resource Efficiency Support

Team, *DLG* Dutch Lymphoedema Guideline, *ISL* International Society for Lymphology, *IUA* International Union of Angiology, *IUP* International Union of Phlebology, *JLSG* The Japan Lymphoedema Study Group, *ILF* Lymphoedema Framework, *NGT* Nominal Group Technique, *ONS* The Oncology Nursing Society, *QH* Queensland Health

Grade A—clear research evidence obtained from meta-analysis or systematic review of randomized controlled trials; Grade B—limited supporting research evidence; and Grade C—experienced common sense judgement (consensus)

Of the eighteen practice points developed, five were generated under lymphoedema prevention, six under assessment, two under lymphoedema diagnosis and four under management.

## Discussion

This consensus process has generated eighteen affordable and feasible practice points to support better lymphoedema care in LMIC. These practice points detail the actions LMIC health care professionals can readily implement to prevent lymphoedema, ensure it is diagnosed early, and manage the condition to prevent or reduce disability and unnecessary suffering and optimise the person’s quality of life. The NGT provided a quick and efficient method of group decision-making and ensured consensus was reached on important topics for practice point generation.

Practice points for prevention require a whole of sector approach to increase community awareness and education regarding lymphoedema, and the importance of meticulous skin care. Drawing on the Ottawa Charter, the three main health promotion strategies required to prevent lymphoedema in LMIC are: 1) advocacy to create the essential conditions to prevent lymphoedema; 2) enabling all people to achieve their full health potential; and 3) mediating between the different interests in society in the pursuit of health [45, 46]. Positive public health policies are needed to increase community awareness of lymphoedema including strengthening community action against lymphoedema [46]. Combatting lymphoedema related stigma requires the creation of an environment free from all forms of prejudice [46].

People at risk of lymphoedema, need to be provided with the skills and resources required to implement simple prevention interventions like meticulous skin care, including hand-hygiene and wearing shoes. A recent meta-analysis of lymphoedema interventional studies found that education focused on hygiene-based techniques in lymphatic filariasis-endemic areas prevented the transmission of lymphatic filariasis through proper hand washing [47], which is equally relevant to people with cancer related and other forms of lymphoedema. For the above actions lymphoedema prevention strategies to be effective, the health systems and services of LMIC require a re-orientation – an effort which should be welcomed by all levels of care (individual, community and health service). Similar to the findings of a previous research on lymphoedema care in LMIC, this NGT consensus identified that increasing community and healthcare workers awareness on lymphoedema was important first step to improving lymphoedema care outcomes in LMIC [26].

Complex decongestive therapy (CDT) is the mainstay of lymphoedema management and is critical to improving function for people living with lymphoedema, regardless of the setting. This involves manual mobilising and drainage of fluid, with meticulous skin care, which ensures a well-moisturised, healthy, clean and intact skin [48].

Practice points for lymphoedema diagnostic techniques generated through our NGT involve comparing circumferential or limb volume measurement of affected and unaffected limbs in unilateral limb lymphoedema or undertaking a baseline measurement of the affected body part. While other diagnostic techniques such as lymphoscintigraphy and Indocyanine green (ICG) florescent lymphography are effective, their prohibitive costs and limited availability makes them unsuitable for use in most LMIC [49]. It is important to promote the less expensive and more straightforward lymphoedema diagnostic methods, such as the use of the volumetric measurement, circumferential measurement as well as palpation in identifying lymphoedema suitable for use in LMIC.

Accurate diagnosis of lymphoedema was one of the major findings of our NGT consensus. Similar to the recommendation of the WHO *Wound and Lymphoedema Management* [13], accurate diagnosis of lymphoedema requires a detailed history, physical examination and correct measurement of limb volumes. Healthcare workers in LMIC should be taught appropriate ways of confirming if 10% of the affected limb is affected by lymphoedema (positive diagnosis) [50] so that they are able to accurately measure and interprete lymphoedema volume measurements.

Practice points generated for lymphoedema assessment include detailed subjective (history taking) and objective assessments. According to Svensson et al. [51], lymphoedema assessment should involve performing a detailed report of presenting signs and symptoms, which were consistent with our study findings that the assessment should include performing Stemmer’s test and physical examination. Despite the bioimpedance spectroscopy is widely known for measuring total fluid volume, our findings indicate that it is expensive, and not readily available for lymphoedema assessment in LMIC.

Another important finding of this study is staging of the lymphoedema. Participants indicated that for proper treatment outcomes, lymphoedema should be classified according to the stages as presented by the International Society of Lymphology [8]. Lymphoedema staging is vital for diagnosing and useful in implementing treatment interventions. A comparative study of the similarities and differences of lymphoedema between HIC and LMIC observed that healthcare workers in both countries are unaware of the disease classification system [14]. It is recommended that healthcare workers will put in efforts to understand the staging system and to implement it in the assessment and diagnosing of lymphoedema in LMIC to improve care for people living with lymphoedema.

In endemic areas of lymphatic filariasis, participants recommended that an ICT be performed. The ICT is a simple and fast method used in detecting *Wuchereria bancrofti* infections responsible for lymphoedema caused by lymphatic filariasis [52]. It is advised that, healthcare workers should request for ICT in cases of podoconiosis to rule out any infection of lymphatic filariasis and to aid early identification and treatment. A review of studies on non-filarial lymphoedema or podoconiosis in Africa indicated that a combination of both laboratory test and geochemical investigations are important in understanding the pathogenesis of the condition, and subsequently developing mechanisms to eradicate lymphoedema [53].

Our NGT confirmed that lymphoedema management in LMIC requires a set of coordinated effort from the multidisciplinary team of healthcare workers. Main management techniques identified in this NGT include meticulous skin care, exercise, modified lymphatic massage, and compression therapy (wraps) based on the stage of the condition. These were not different from CDT, the key management strategy for lymphoedema [31]. The main difference in LMIC is that specialist skills may be limited, and some resources may not be available for its implementation. Based on this factor, experts of our NGT suggest the use of improvised materials such as the wraps and simple treatment modalities. Additionally, this NGT consensus indicated that the management of lymphoedema should involve proper foot and skin hygiene, and wearing of appropriate foot wears in podoconiosis endemic areas. Where appropriate, lymphoedema treatment should include pelvic floor muscle exercises for people living with genital lymphoedema.

Despite the importance of surgical procedures such as lymphaticovenular anastomosis or vascularised lymph node transfer which increase limb volumes [54], these procedures require well-trained surgeons and experienced healthcare workers which are inaccessible in LMIC due to a lack of suitable facilities or cost. It is hoped that, with the advance of technology and health systems globally, LMIC will be able to secure appropriate facilities and skills to help optimise conditions for surgery. A considerable strength of these lymphoedema care practice points are the use of an international multi-disciplinary consensus process and the development of guidance that is cost-effective and feasible for all health professionals in LMIC to apply.

## Limitations

Our inability to identify and include a larger number of healthcare workers providing lymphoedema care in LMIC is a limitation. However, the participants who contributed were lymphoedema experts with diverse experience across most LMIC regions.

Another limitation was not specifying specific management for various etiologies including primary, genetic or secondary lymphoedema. Instead, we provided practice points suitable for treating all kinds of lymphoedema no matter the stage of the condition. Further research should consider providing targeted treatments for genetic and non-genetic causes of lymphoedema. Finally, there is likely to have been a sampling bias and we were not able to ascertain reasons for non-response to invitations to take part in the NGT.

## Implications for future research

Ideally, the practice points developed through the consensus process reported in this paper should be evaluated in research, with the gold standard being randomised controlled trials.

Implementation of these practice points will also need a concerted effort that should be underpinned by research aimed at evaluating their impacts and informing similar efforts for other guidance in the future. According to the Guidelines International Network, new guidelines should be accompanied by campaigns aimed at raising awareness, taking into account cultural and social contexts of the LMIC in which they will be implemented. Practice points should also be made readily accessible to all healthcare workers through open access journals and websites of non-governmental agencies that focus on LMIC healthcare delivery.

Lymphoedema education for healthcare workers could not be underestimated in the prevention of lymphoedema in LMIC. However, most healthcare workers lack appropriate knowledge and professional information required for lymphoedema management of people living with lymphoedema. It is appropriate that, in-service training and structured workshops with focus on increasing healthcare workers knowledge on lymphoedema be instituted to support care in LMIC.

## Conclusion

Healthcare workers require increased knowledge and awareness to improve lymphoedema care in LMIC. Eighteen practice points have been developed to optimise lymphoedema care in LMIC. Key lymphoedema care strategies identified in this NGT consensus include meticulous skin care, exercise, modified lymphatic massage, and use of compression bandages or wraps. These practice points require dissemination to enhance lymphoedema care and improve clinical practice on management in LMIC.

## Supplementary Information

**Additional  file 1.** 

## Data Availability

The datasets analysed in the current study are available from the corresponding author on reasonable request.

## Abbreviations

ACI: Agency for Clinical Innovation
APTA: American Physical Therapy Association
BPG: Best Practice Guideline
CDT: Complex Decongestive Therapy
CREST: Clinical Resource Efficiency Support Team
DLG: Dutch Lymphoedema Guideline
HIC: High-Income Countries
ICG: Indocyanine Green
ICT: Immunochromatographic Card Test
ILF: International Lymphoedema Framework
ISL: International Society for Lymphology
IUA: International Union of Angiology
IUP: International Union of Phlebology
JLSG: The Japan Lymphoedema Study Group
LMIC: Low- and Middle-Income Countries
NGT: Nominal Group Technique
ONS: The Oncology Nursing Society
QH: Queensland Health
WHO: World Health Organisation

## References

[1] Adamczyk LA, Gordon K, Kholová I, Meijer-Jorna LB, Telinius N, Gallagher PJ (2016). Lymph vessels: the forgotten second circulation in health and disease. Virchows Archiv.

[2] Maree JE, Beckmann D (2016). “Just live with it”: Having to live with breast cancer related lymphoedema. Health SA Gesondheid.

[3] Son A, O'Donnell TF, Izhakoff J, Gaebler JA, Niecko T, Iafrati MA (2019). Lymphoedema-associated comorbidities and treatment gap. J Vasc Surg Venous Lymphat Disord.

[4] Földi M, Földi E, Strößenreuther C, Kubik S. Földi's textbook of lymphology: for physicians and lymphedema therapists: Elsevier Health Sciences; 2012.

[5] Cromwell KD, Chiang Y-J, Armer J, Heppner PP, Mungovan K, Ross MI (2015). Is surviving enough? Coping and impact on activities of daily living among melanoma patients with lymphoedema. Eur J Cancer Care.

[6] Meiklejohn JA, Heesch KC, Janda M, Hayes SC (2013). How people construct their experience of living with secondary lymphoedema in the context of their everyday lives in Australia. Support Care Cancer.

[7] Schulze H, Nacke M, Gutenbrunner C, Hadamitzky C (2018). Worldwide assessment of healthcare personnel dealing with lymphoedema. Heal Econ Rev.

[8] International Society of Lymphology (2016). The diagnosis and treatment of peripheral lymphedema: 2016 consensus document of the international society of lymphology. Lymphology.

[9] Deshpande A, Miller-Petrie MK, Lindstedt PA, Baumann MM, Johnson KB, Blacker BF (2020). The global distribution of lymphatic filariasis, 2000–18: a geospatial analysis. Lancet Glob Health.

[10] World Health Organisation. Lymphatic filariasis 2021 [cited 2021 19 July]. Available from: https://www.who.int/news-room/fact-sheets/detail/lymphatic-filariasis. Accessed 19 July 2021.

[11] WHO. Lymphatic filariasis: reporting continued progress towards elimination as a public health problem Geneva2020 [Available from: https://www.who.int/news/item/29-10-2020-lymphatic-filariasis-reporting-continued-progress-towards-elimination-as-a-public-health-problem. Accessed 22 Nov 2021.

[12] O'Donnell Jr TF, Allison GM, Iafrati MD. A systematic review of guidelines for lymphedema and the need for contemporary intersocietal guidelines for the management of lymphedema. J Vasc Surg Venous Lymphat Disord. 2020. 10.1016/j.jvsv.2020.03.006 32444277

[13] World Health Organisation. Wound and lymphoedema management. Geneva2010. Available from: https://www.who.int/lymphatic_filariasis/resources/9789241599139/en/. Accessed 9 June 2020.

[14] Stout NL, Brantus P, Moffatt C. Lymphoedema management: an international intersect between developed and developing countries. Similarities, differences and challenges. Global Public Health. 2012;7(2):107–23. 10.1080/17441692.2010.549140 21360379

[15] Tesfaye A, Semrau M, Ali O, Kinfe M, Tamiru M, Fekadu A (2021). Development of an integrated, holistic care package for people with lymphoedema for use at the level of the Primary Health Care Unit in Ethiopia. PLoS Negl Trop Dis.

[16] Cancer Australia. Recommendations and practice points 2022 [Available from: https://www.canceraustralia.gov.au/resources/clinical-practice-guidelines/first-line-chemotherapy-treatment-women-epithelial-ovarian-cancer/recommendations-and-practice-points. Accessed 5 June 2020.

[17] Harvey N, Holmes CA (2012). Nominal group technique: an effective method for obtaining group consensus. Int J Nurs Pract.

[18] Rankin NM, McGregor D, Butow PN, White K, Phillips JL, Young JM (2016). Adapting the nominal group technique for priority setting of evidence-practice gaps in implementation science. BMC Med Res Methodol.

[19] MacPhail A (2001). Nominal group technique: a useful method for working with young people. Br Edu Res J.

[20] Irving MJ, Jan S, Tong A, Wong G, Craig JC, Chadban S (2014). What factors influence people's decisions to register for organ donation? The results of a nominal group study. Transpl Int.

[21] Redman S, Carrick S, Cockburn J, Hirst S (1997). Consulting about priorities for the NHMRC National Breast Cancer Centre: how good is the nominal group technique. Aust N Z J Public Health.

[22] The World Bank Group. World Bank Country and Lending Groups 2019 [Available from: https://datahelpdesk.worldbank.org/knowledgebase/articles/906519-world-bank-country-and-lending-groups. Accessed 17 June 2019.

[23] Marcus B, Weigelt O, Hergert J, Gurt J, Gelléri P (2017). The use of snowball sampling for multi source organizational research: Some cause for concern. Pers Psychol.

[24] Sharma G (2017). Pros and cons of different sampling techniques. Int J Appl Res.

[25] Torgbenu E, Luckett T, Buhagiar MA, Chang S, Phillips JL (2020). Prevalence and incidence of cancer related lymphedema in low and middle-income countries: a systematic review and meta-analysis. BMC Cancer.

[26] Torgbenu E, Luckett T, Buhagiar M, Requena CM, Phillips JL (2022). Improving care for cancer-related and other forms of lymphoedema in low-and middle-income countries: a qualitative study. BMC Health Serv Res.

[27] Meho LI (2006). E-mail interviewing in qualitative research: A methodological discussion. J Am Soc Inform Sci Technol.

[28] McMillan SS, King M, Tully MP (2016). How to use the nominal group and Delphi techniques. Int J Clin Pharm.

[29] Morgan P, Moffatt C (2006). An update on the Lymphoedema Framework Project. Br J Community Nurs.

[30] Armer JM, Ostby PL, Ginex PK, Beck M, Deng J, Fu MR (2020). ONS guidelines™ for Cancer Treatment-Related Lymphedema. Oncol Nurs Forum.

[31] Gebruers N, Verbelen H, De Vrieze T, Vos L, Devoogdt N, Fias L (2017). Current and future perspectives on the evaluation, prevention and conservative management of breast cancer related lymphoedema: A best practice guideline. Eur J Obstet Gynecol Reprod Biol.

[32] McMillan SS, Kelly F, Sav A, Kendall E, King MA, Whitty JA (2014). Using the nominal group technique: how to analyse across multiple groups. Health Serv Outcomes Res Method.

[33] Doke ER, Swanson NE (1995). Decision variables for selecting prototyping in information systems development: a Delphi study of MIS managers. Inform Manag.

[34] Holey EA, Feeley JL, Dixon J, Whittaker VJ (2007). An exploration of the use of simple statistics to measure consensus and stability in Delphi studies. BMC Med Res Methodol.

[35] The AGREE II Instrument [Electronic version]. [Internet]. 2017 [cited 10 October 2020]. Available from: http://www.agreetrust.org. Accessed 10 Oct 2020.

[36] Agency for Clinical Innovation Chronic Care Network. Lymphoedema: A guide for clinical services 2018 [Available from: https://www.aci.health.nsw.gov.au/__data/assets/pdf_file/0008/477998/lymphoedema-guide.pdf. Accessed 30 Jan 2020.

[37] Lee BB, Andrade M, Antignani PL, Boccardo F, Bunke N, Campisi C, et al. Diagnosis and treatment of primary lymphedema. Consensus document of the International Union of Phlebology (IUP)-2013. Int Angiol. 2013;32(6):541–74. 24212289

[38] Perdomo M, Ryans K, Levenhagen K, Davies CC, Gilchrist L (2018). Clinical implementation of the clinical practice guidelines for diagnosing upper-quadrant lymphedema secondary to cancer. Rehab Oncol.

[39] Lee BB, Antignani PL, Baroncelli TA, Boccardo FM, Brorson H, Campisi C (2015). IUA-ISVI consensus for diagnosis guideline of chronic lymphedema of the limbs. Int Angiol.

[40] Clinical Resource Efficiency Support Team. Guidelines for the diagnosis, assessment and management of lymphoedema: Clinical Resource Efficiency Support Team; 2008.

[41] Manji K, Hanefeld J, Vearey J, Walls H, de Gruchy T (2021). Using WhatsApp messenger for health systems research: a scoping review of available literature. Health Policy Plan.

[42] Queensland Health. Queensland Health lymphoedema clinical practice guideline 2014 [Available from: https://www.health.qld.gov.au/__data/assets/pdf_file/0027/146646/guideline-lymph.pdf.

[43] Davies C, Levenhagen K, Ryans K, Perdomo M, Gilchrist L (2020). Interventions for breast cancer-related lymphedema: clinical practice guideline from the academy of oncologic physical therapy of APTA. Phys Ther.

[44] Kitamura K, Iwase S, Kuroda Y, Yamaguchi T, Yamamoto D, Odagiri H (2011). A practice guideline for the management of lymphoedema. J Lymphoedema.

[45] Thompson SR, Watson MC, Tilford S (2018). The Ottawa Charter 30 years on: still an important standard for health promotion. Int J Health Promot Educ.

[46] World Health Organization (1986). Ottawa charter for health promotion. Health Promot.

[47] Stocks ME, Freeman MC, Addiss DG (2015). The effect of hygiene-based lymphedema management in lymphatic filariasis-endemic areas: a systematic review and meta-analysis. PLoS Negl Trop Dis.

[48] Nowicki J, Siviour A (2013). Best practice skin care management in lymphoedema. Wound Pract Res J Austral Wound Manag Assoc.

[49] Mahieu R, Krijger GC, Ververs FT, de Roos R, de Bree R, de Keizer B. [68 Ga] Ga-tilmanocept PET/CT lymphoscintigraphy: a novel technique for sentinel lymph node imaging. Eur J Nucl Med Mol Imaging. 2021;48:963–5. 10.1007/s00259-020-05101-5 33159222

[50] Cheville AL, Nyman JA, Pruthi S, Basford JR (2012). Cost considerations regarding the prospective surveillance model for breast cancer survivors. Cancer.

[51] Svensson BJ, Dylke ES, Ward LC, Black DA, Kilbreath SL (2020). Screening for breast cancer–related lymphoedema: self-assessment of symptoms and signs. Support Care Cancer.

[52] Weil GJ, Lammie PJ, Weiss N (1997). The ICT filariasis test: a rapid-format antigen test for diagnosis of bancroftian filariasis. Parasitol Today.

[53] Wanji S, Deribe K, Minich J, Debrah AY, Kalinga A, Kroidl I (2021). Podoconiosis-From known to unknown: Obstacles to tackle. Acta Trop.

[54] Phillips GSA, Gore S, Ramsden A, Furniss D (2019). Lymphaticovenular anastomosis in the treatment of secondary lymphoedema of the legs after cancer treatment. J Plast Reconstr Aesthet Surg.

