# Supplementary material for: Practice points for lymphoedema care in low- and middle- income countries developed by nominal group technique
Source: BMC Health Serv Res. 2023 Jul 8;23:740. doi: 10.1186/s12913-023-09786-w (PMC10329783; doi:10.1186/s12913-023-09786-w)
Supplement: Supplementary file 1 — Additional file 1. [file 12913_2023_9786_MOESM1_ESM.docx]

Supplementary Table 1: Lymphoedema organisations and their web pages

| **Lymphoedema Organisations** | **Abbreviations** | **Web pages** |
| --- | --- | --- |
| Agency for Clinical Innovation | ACI | https://aci.health.nsw.gov.au/ |
| American Physical Therapy Association | APTA | https://www.apta.org/ |
| Best Practice Guideline | BPG | https://www.ejog.org/ |
| Clinical Resource Efficiency Support  Team | CREST | https://www.lymphoedemasupportni.org/news/clinical-efficiency-support-team-crest |
| Dutch Lymphoedema Guideline | DLG | https://vascern.eu/actualite/dutch-lymphedema-guidelines/ |
| International Society for Lymphology | ISL | https://www.italf.org/2016-consensus-document-of-the-international-society-of-lymphology/ |
| International Union of Angiology | IUA | https://uia.org/s/or/en/1100053222 |
| International Union of Phlebology | IUP | https://www.uip-phlebology.org/ |
| The Japan Lymphoedema Study Group | JLSG | https://woundsinternational.com/journal-of-lymphoedema/ |
| International Lymphoedema Framework | ILF | https://www.lympho.org/index.php |
| The Oncology Nursing Society | ONS | https://www.ons.org/ |
| Queensland Health | QH | https://www.health.qld.gov.au/__data/assets/pdf_file/0027/146646/guideline-lymph.pdf |
